# Methodological issues of the electronic health records’ use in the context of epidemiological investigations, in light of missing data: a review of the recent literature

**DOI:** 10.1186/s12874-023-02004-5

**Published:** 2023-08-09

**Authors:** Thomas Tsiampalis, Demosthenes Panagiotakos

**Affiliations:** 1 Department of Nutrition and Dietetics, School of Health Sciences and Education, Harokopio University, Athens, Greece; 2 Faculty of Health, University of Canberra, Canberra, Australia

**Keywords:** Electronic health record, Healthcare quality, Medical decision, Missing data, Save cost and time

## Abstract

**Background:**

Electronic health records (EHRs) are widely accepted to enhance the health care quality, patient monitoring, and early prevention of various diseases, even when there is incomplete or missing information in them.

**Aim:**

The present review sought to investigate the impact of EHR implementation on healthcare quality and medical decision in the context of epidemiological investigations, considering missing or incomplete data.

**Methods:**

Google scholar, Medline (via PubMed) and Scopus databases were searched for studies investigating the impact of EHR implementation on healthcare quality and medical decision, as well as for studies investigating the way of dealing with missing data, and their impact on medical decision and the development process of prediction models. Electronic searches were carried out up to 2022.

**Results:**

EHRs were shown that they constitute an increasingly important tool for both physicians, decision makers and patients, which can improve national healthcare systems both for the convenience of patients and doctors, while they improve the quality of health care as well as they can also be used in order to save money. As far as the missing data handling techniques is concerned, several investigators have already tried to propose the best possible methodology, yet there is no wide consensus and acceptance in the scientific community, while there are also crucial gaps which should be addressed.

**Conclusions:**

Through the present thorough investigation, the importance of the EHRs’ implementation in clinical practice was established, while at the same time the gap of knowledge regarding the missing data handling techniques was also pointed out.

## Introduction

Electronic Health Records (EHRs) constitute a challenging information system including a big, valuable collection of health information about patients’ medical history and other related characteristics, both in structured and unstructured format. EHR have been implemented by an ever-increasing number of hospitals and research institutions around the world, as the mobile computing has been grown tremendously and the number of records regarding personal health has been increasing exponentially [1]. According to the US Health Information Technology for Economic and Clinical Health Act (HITECH Act), in 2009, a spending exceeding $30 billion was authorized for the EHR adoption [2], with the EHR installations having been increased tremendously,between 2010 and 2014, the number of hospitals with a basic EHR system rose from 15.6% to 75.5% [3]. By 2025, the European Commission is looking to digitize all medical records throughout the 27-member bloc of European Union, to make it easier for individuals to access and share their personal data with medical professionals, particularly when they are in another country [4]. Moreover, EHR constitute a cornerstone of what is now called Real World Data, but this is a topic for another methodological review.

Several studies have already highlighted that EHRs may sufficiently improve the quality of healthcare, increase time efficiency and guideline adherence, and reduce medication errors and adverse drug effects [5–8]. At the same time, the use of EHRs in the medical decision process is rapidly growing, with an increasing number of researchers using them for the prognosis and early diagnosis of various chronic and non-chronic diseases [9]. An emerging literature has already recognized the challenges that still lay ahead in using EHRs’ data in epidemiological research. The most crucial issue is the population representativeness included in EHRs (i..e, revealing the issue of selection bias), as well as the missing information in crucial clinical measurements and outcomes [10–14]. These issues are considered to be inevitable in real-world studies [15, 16], as their existence could be attributed to several reasons (e.g., refusal of patients to answer sensitive questions, lost- to follow- up, etc.). According to Bell et al., [17], as well as Little and Rubin [18], this can also lead to a substantial decrease in the efficiency and validity of the conducted data analyses and therefore, distort inferences about the referent population. Therefore, it is of crucial importance to identify the profile of the individuals with missing data, as well as to implement the right methodological approach, so as to impute the missing data and derive efficient and valid conclusions [19, 20].

The aim of the present review is to present the challenges faced during the use of the EHRs for epidemiological investigations in the context of missing data, as well as to discuss the most frequent statistical methodologies being implemented for handling such cases and confronting the obstacle of missing information to derive valid conclusions.

## Material and methods

### Eligibility criteria

#### Type of studies

The present review has been conducted according to the Preferred Reporting Items for Systematic Reviews and Meta-Analyses (PRISMA; [21]). Case studies, cohort studies, cross-sectional studies, retrospective case–control, prospective cohort, and cluster-randomized controlled trials, published in English language, either conducted in a hospital setting or not, were included in the present review, while systematic reviews and meta-analyses were excluded (but assisted in retrieving articles not allocated in search process).

### Information sources and search strategy

Relevant studies, without any chronological and country restriction, were identified by searching in Medline (via PubMed), Scopus, and Google scholar databases by using the search strategies presented in Table 1. After removing the duplicate studies found among the different databases, articles were manually and independently screened by both authors (TT, DP), based on their Title and Abstract and then full text reading was conducted for the final selection decision. In the case of disagreement, another scientist was asked to comment on the eligibility of the reviewed study.

**Table 1:** Search strategies in each database for retrieving the most appropriate research works

Database	Search strategy
PubMed	**Search strategy 1-** ((((“electronic health records”) AND (healthcare quality)) AND (save)) AND (improve)) AND (patients); **Search strategy 2**- (((electronic health record) AND ((medical OR healthcare) AND decision) AND (missing data)) NOT (impact)) NOT (systematic review); **Search strategy 3**-((((“electronic health records missing data imputation”) NOT (systematic review)) NOT (meta-analysis)) NOT (review))
Scopus	**Search strategy 1-** TITLE-ABS-KEY (“electronic health records” AND ((healthcare OR medical) AND (choice OR process OR decision OR order)) AND ((lost OR missing) AND data)); **Search strategy 2-** TITLE-ABS-KEY (“electronic medical records” AND ((healthcare OR medical) AND (choice OR process OR decision OR order)) AND ((lost OR missing) AND data) AND NOT (impact OR drawback)) AND (LIMIT-TO (SUBJAREA, “MEDI”)); **Search strategy 3-** TITLE-ABS-KEY (“electronic health records” AND “missing data” AND (imputation OR generation) AND NOT (systematic AND review OR review)); **Search strategy 4-** electronic health record AND healthcare quality AND save cost time AND health system AND improve AND NOT systematic review AND NOT meta-analysis
Google Scholar	**Search strategy 1-** (“benefits” OR “advantage”)(“electronic health records”)(“healthcare quality”)(“medical decision” OR “medical choice””) (“missing data”); **Search strategy 2-** EHRs missing data medical decision quality advantage OR benefits “electronic health records” “missing data” -impact -affect –“systematic review”; **Search strategy 3-** electronic health records missing data imputation “electronic health records” -impact -drawback –“systematic review” -review –“meta analysis”

## Results

### Study selection

Of the 1972 references initially identified from the electronic and manual search studies (PubMed: 313; Scopus: 519; Google scholar: 1140), a total of 17 studies were included in the present narrative review, which were divided in two categories:

i) studies related to the benefits of the EHRs implementation on medical quality and health system (e.g., cost- savings, reduced medical errors, improved emergency care etc.)
ii) studies related to the methodologies being implemented for imputing missing data in the context of the EHRs.

At first, 20 duplicate records were removed, and then the remaining 1,952 records were screened based on their title and abstract. From those, 1,897 records were removed due to irrelevance to the aim of the present review. Finally, 38 records were also removed as we were not able to retrieve them from the authors after contacting them (i.e., *not available in full- text*). Thus, in category 1, 8 studies were reviewed, and in category 2, 9 studies were reviewed. In Table 2 the selection process of the studies is described.

**Table 2:** Selection process of the studies included in the review

	**Number of records**
**Total records identified:**	**1,972**
*PubMed*	*313*
*Scopus*	*519*
*Google Scholar*	*1,140*
**Duplicate records among different databases**	**20**
**Records screened (Title & Abstract) after duplicate removal**	**1,952**
**Total records excluded:**	**1,935**
*Due to irrelevance*	*1,897*
*Due to inability to retrieve them from the authors*	*38*
**Total records included in the review**	**17**
*‘EHRs and improvement of medical quality and health system’*	*8*
*‘Missing data in the context of EHRs’*	*9*

### EHRs and quality, in relation to medical decision making

In a case study published by Vuppalapati et al., [22] it was shown that selfies constitute important outpatient healthcare data which could improve the diagnosis of diseases, as well as the decision-making process. More specifically, it was reported that selfies taken for medical image purposes constitute valuable outpatient healthcare data providing new clinical insights, while they could also be used as diagnostics markers for the provision of prognosis of potential masked diseases. In addition, according to Bar-Dayan et al., [23], whose main aim was to assess the effectiveness of using the EHRs in terms of cost-savings, EHRs were shown to yield significant improvements, both to physicians, as well as to clinic practices and healthcare organizations, as they were shown to provide substantial cost- savings.

Electronic health records can assist in both the prevention, as well as the treatment of a disease. Lardon et al., [24] based on EHR data, developed rules to support diagnosis coding of chronic kidney disease (CKD) in the hospital of Saint Etienne. In another study of of Garnica et al., [25] electronic health records were shown to help in the prognosis of bacteremia, involving early diagnosis for the provision of treatments to avoid complications and death. Machine Learning (ML) techniques were applied to predict the result of blood culture for the timely administration of the correct treatment thus reducing medical costs. Furthermore, Zaballa et al., [26] presented a general framework to identify and discover the most common treatment pathways which are being exploited to treat diseases. Besides, King et al., [27] confirmed the clinical benefits of EHRs through cross-sectional data examination. EHR adopters reported benefits of EHR use in terms of clinical quality, patient safety, and efficiency, while the use of an EHR meeting *Meaningful Use* criteria was found to be significantly associated with reporting clinical benefits enabled by these functionalities. Except for that, as claimed by Huang et al., [28] EHRs constitute valuable tools which can help in the prediction of multi-type major adverse cardiovascular events. According to Linder et al., [29] it was also shown that EHR–based interventions can improve the smoking status documentation and increase the counseling assistance to smokers. In Table 3 the main findings regarding the contribution of the EHRs on medical quality and the health system, are presented.

**Table 3:** Main findings regarding the contribution of Electronic Health Records on the improvement of medical quality and health system

Study (Author, Year)	Main findings
Vuppalapati et al. [22]	- Selfies taken for medical image purposes are valuable outpatient healthcare data assets that could provide new clinical insights - Diagnostics markers that could provide prognosis of a potential masked disease and necessitate actions to avert any emergency incidence - Improve overall health outcomes of people around the globe in a cost-effective manner that epitomizes the confluence of popularity with curiosity and sharing with accountability
Bar-Dayan et al. [23]	- Positive net financial return from using an electronic medical record system - Referring patients to preferred providers from classes 1–3 was achieved without administrative staff aid. Increased efficiency by redirecting the administrative manpower engaged in this task to other goals - Using EHRs to direct referrals to preferred specialty care physicians, accompanied by a comprehensive physician education program, can play a significant role in facilitating effective utilization of healthcare providers and in lowering costs
Lardon et al. [24]	- A business rule management system could be a good basis to implement a tool to help and check diagnosis codes - Development of rules based on EHR data with the Drools rules engine in order to support diagnosis coding of chronic kidney disease (CKD)
Garnica et al. [25]	- Electronic health records were shown to help in the prognosis of bacteremia, involving early diagnosis for the provision of treatments to avoid complications and death - The three ML supervised classifiers create accurate predictive models of the blood culture outcome using hospital electronic health records
Zaballa et al. [26]	- Identification of the actions in the health system associated with a disease - Identification of those patients with a complete treatment for the disease - Discovery of common treatment pathways followed by the patients with a specific diagnosis
King et al. [27]	- Most physicians with EHRs reported EHR use enhanced patient care overall, helped them access a patient’s chart remotely, and alerted them to a potential medication error and critical lab values - EHR use was associated with clinical benefits related to providing recommended care, ordering appropriate tests, and facilitating patient communication
Huang et al. [28]	- Timely and accurate prediction of major adverse cardiovascular events after acute coronary syndrome - Assist clinicians to pay more attention to high-risk patients and improve the quality and efficiency of care accordingly
Linder et al. [29]	- This electronic health record–based intervention improved smoking status documentation and increased counseling assistance to smokers - There was also a suggestion that outcomes of care may have also improved

### Missing data in the context of EHRs

In the context of EHRs, lack of documentation is mainly observed in cases when the patients do not have a symptom or comorbidity. In these cases, instead of recording a negative value for each potential symptom/comorbidity, all data fields are left missing and only the positive values are recorded. Therefore, lack of a symptom/comorbidity, lack of documentation of a symptom/comorbidity and lack of data collection regarding the symptom/comorbidity cannot be differentiated.

According to the reviewed literature, there is a variety of approaches toward managing missing EHR data; Goldstein et al., [30], who conducted a systematic review regarding the challenges faced during the development of risk prediction models based on EHRs, found that only 58 of the 90 studies (64%) evaluated addressed missing data prior to analysis. Some of the simplest methodological approaches being used, involve the selection of sub-datasets that contain complete information [31, 32], as well as the stratified mean imputation [33], while others have advanced statistical methodologies which are applicable only to continuous measures and interpolate longitudinal variables with limited individual-level variability that are typically not dependent on other covariates [34]. Despite these approaches, few studies utilized “*informative observations*” where the presence of a variable is meaningful for the possibly missing values [30]. Xu et al., [35] developed a deep learning unsupervised method to impute missing values in patient records and by comparing it with four other imputation techniques, they showed that the specific methodology could significantly reduce the imputation biases under various scenarios, and as a result it could empower physicians and researchers to better utilize the EHRs aiming at improved patient management.

**Fig. 1:**
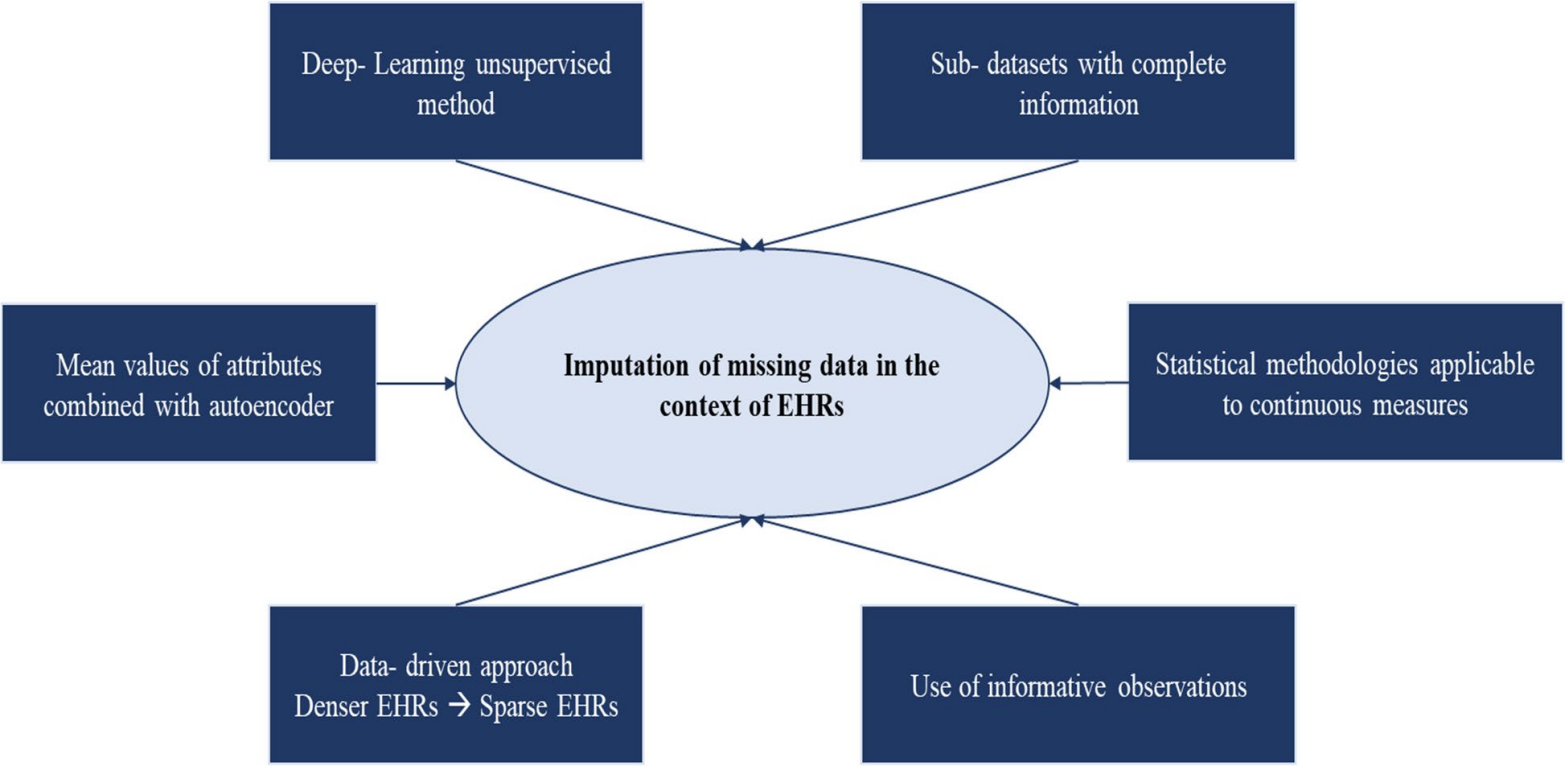
Missing data imputation techniques in the context of EHRs, based on the research works included in the present review

In addition, Hwang, et al. [36] proposed a two-stage framework leading to more robust results for disease prediction based on EHRs with missing data. Two different imputation methods were implemented, the first of which replaced the missing values with the mean values of the attributes, while the second one used an autoencoder, which is an unsupervised ML algorithm. Furthermore, Wang et al. [37], based on the idea that among heterogeneous patient populations there exist homogeneous groups of patients, proposed a data driven approach for imputing the sparse patient EHRs by transferring relevant knowledge from patients with denser EHRs to their patients with sparse EHRs. In Fig. 1 an overview of the methodologies used for imputing missing data in the context of the EHRs, based on the research works included in the present review, is illustrated.

## Discussion

Based on the present review, EHRs constitute an increasingly important tool for both healthcare professionals and decision makers, which can improve national healthcare systems both for the convenience of patients and doctors, by helping on the prevention and treatment of chronic and non- chronic diseases, while regarding the statistical methodologies being implemented for imputing missing data, further steps should be conducted and new methodologies should be proposed and be tested in this context.

### Benefits of EHRs

As already pointed out, some of the most important benefits related to EHRs include the easy access to computerized records, as well as the elimination of poor penmanship, which constitutes a widespread and significant obstacle in the medical world [38, 39]. Besides, EHRs provide significant cost savings, as based on the studies of Shu et al. [40] and Bar- Dayan et al. [23], it was shown that the release of EHR data to patients via smart apps can save both the hospital, as well as the patients, approximately 2 million and 1 million euros, respectively, on an annual basis. This could be attributed to the fact that, the EHR’s use can substantially reduce the redundant implementation of medical tests or the need to mail hard copies of test results to different providers [41, 42]. Additionally, several studies have also shown that EHRs, compared to hard- copies, result in reduced transcription costs through point-of-care documentation and other structured documentation procedures [43]. Furthermore, the access to electronically stored data increases the availability of data, which leads to the improvement of the ability to conduct research, as well as to the facilitation of the identification of evidence- based best health practices [44], while at the same time public health researchers by using EHRs tend to produce more beneficial for the society research outcomes. Even more, according to several studies, despite the fact that EHRs have known drawbacks when they are used solely as data sources for studies informing public health decisions [45], they contain several crucial data elements which help with a pandemic response [46, 47].

### Missing data handling techniques

As far as the missing data handling techniques is concerned, several investigators have already tried to propose the best possible methodology, yet there is no wide consensus and acceptance in the scientific community, while there are also crucial gaps which should be addressed. As pointed out, missing information constitutes a widely spread phenomenon in routinely collected health data and often missingness is very informative and should be incorporated into the development process of prediction and epidemiological models [48, 49], as the absence of data in EHR records can substantially decrease our ability to create accurate predictions [49]. Besides, the majority of the hitherto developed prediction models are not able to provide a risk estimate when missing information exist in predictor variables, which delays their implementation and may ultimately limit guideline adherence [50]. However, the correct way of handling missing values particularly in the phase of prediction model development and in the validation dataset, solely depends on the intended use of the prediction model, and more specifically, on whether the investigator intends to allow for missing data during model application in practice [51]. So far, in clinical practise and in a real clinical setting, when applying already developed prediction models in new patients arising in the medical office to predict their risk of disease onset or disease recurrence, accounting for missing values in some of their demographic or clinical characteristics is not straightforward. Ideally, when developing a prediction model the methodology regarding the handling of missing data should be integrated, however this is not a usual case in practise, as most of the developed models do not allow for missing data [51–63].

#### Limitations of the literature review process

However, this review paper has some limitations, such as the fact that there is not a well-established metric to evaluate the performance of the EHRs in clinical practice. Therefore, no quantitative assessment could be performed that also evaluate the cost-effectiveness of EHR in medical decision making. Moreover, no pooled analysis or quality assessment of the reviewed studies was performed, as this was out of the scope of the present work, and in many cases was not feasible.

## Conclusions

Despite the limitations of the present review, the importance of the EHRs’ implementation in clinical practice was highlighted, while at the same time the gap of knowledge regarding the missing data handling techniques was also pointed out. EHRs seems that they constitute an increasingly important tool for both physicians, decision makers and patients, which can improve national healthcare systems both for the convenience of patients and doctors, while they improve the quality of health care as well as they can also be used to save money.

## Data Availability

Not applicable.

## Abbreviations

CKD: Chronic Kidney Disease
EHRs: Electronic Health Records
ML: Machine–Learning
PRISMA: Preferred Reporting Items for Systematic Reviews and Meta-Analyses
